# Multiple pathways regulate shoot branching

**DOI:** 10.3389/fpls.2014.00741

**Published:** 2015-01-13

**Authors:** Catherine Rameau, Jessica Bertheloot, Nathalie Leduc, Bruno Andrieu, Fabrice Foucher, Soulaiman Sakr

**Affiliations:** ^1^Institut Jean-Pierre Bourgin, INRA, UMR 1318, ERL CNRS 3559, Saclay Plant Sciences, Versailles, France; ^2^Institut Jean-Pierre Bourgin, AgroParisTech, UMR 1318, ERL CNRS 3559, Saclay Plant Sciences, Versailles, France; ^3^UMR1345 IRHS, INRA, SFR 4207 QUASAV, Beaucouzé, France; ^4^UMR1345 IRHS, Université d’Angers, SFR 4207 QUASAV, Angers, France; ^5^UMR1091 EGC, INRA, Thiverval-Grignon, France; ^6^UMR1091 EGC, AgroParisTech, Thiverval-Grignon, France; ^7^UMR1345 IRHS, Agrocampus-Ouest, SFR 4207 QUASAV, Angers, France

**Keywords:** axillary bud outgrowth, apical dominance, polar auxin transport, strigolactone, cytokinins, shade avoidance, flowering, modeling

## Abstract

Shoot branching patterns result from the spatio-temporal regulation of axillary bud outgrowth. Numerous endogenous, developmental and environmental factors are integrated at the bud and plant levels to determine numbers of growing shoots. Multiple pathways that converge to common integrators are most probably involved. We propose several pathways involving not only the classical hormones auxin, cytokinins and strigolactones, but also other signals with a strong influence on shoot branching such as gibberellins, sugars or molecular actors of plant phase transition. We also deal with recent findings about the molecular mechanisms and the pathway involved in the response to shade as an example of an environmental signal controlling branching. We propose the TEOSINTE BRANCHED1, CYCLOIDEA, PCF transcription factor TB1/BRC1 and the polar auxin transport stream in the stem as possible integrators of these pathways. We finally discuss how modeling can help to represent this highly dynamic system by articulating knowledges and hypothesis and calculating the phenotype properties they imply.

## INTRODUCTION

The pattern of shoot branching, a major component of plant architecture, results from a complex spatio-temporal regulation of axillary bud outgrowth. Axillary meristems initiated at the axils of most leaves initiate a few leaves to form an axillary bud. These buds can undergo immediate growth and turn into a lateral branch or become dormant. Dormancy is not definitive: the bud can often resume its growth, e.g., in case of damage to the apex or at flowering initiation (Stafstrom and Sussex, 1988; Shimizu-Sato and Mori, 2001; Beveridge et al., 2003; McSteen and Leyser, 2005). At the level of each axillary bud and at the plant level, many endogenous and developmental signals have to be integrated to determine bud fate and to establish the number and position of the growing new shoots on the plant. Such regulation is also strongly dependent on environmental factors (Khayat and Zieslin, 1982; Moulia et al., 1999; Battey, 2000; Cameron et al., 2006; Kim et al., 2010; Huché-Thélier et al., 2011; Demotes-Mainard et al., 2013; Djennane et al., 2014; Pierik and Testerink, 2014), so that plants adjust their branching capacity according to the environmental conditions they are submitted to. Among these environmental factors, light is a major factor (Leduc et al., 2014); plants modulate bud outgrowth and branch development according to the light parameters they sense, i.e., light intensity—as expressed by the photosynthetic photon flux density (PPFD); light quality—depending on wavelengths and their relative proportions; and the photoperiod—the respective amounts of light and dark in a daily cycle of 24 h (Jackson, 2009). Here we considered recent molecular and biochemical results suggesting the involvement of different pathways in the control of axillary bud outgrowth and their complex interactions. We did not address axillary meristem initiation or branching of the inflorescence, as they involve other gene networks and other processes than vegetative bud outgrowth (Schmitz and Theres, 2005). These processes have been reviewed recently (Janssen et al., 2014; Teo et al., 2014)

For decades, the study of shoot branching has been based on decapitation experiments where removal of the shoot apex of a growing shoot stimulates the outgrowth of axillary buds. The term apical dominance was proposed for this inhibitory role of the shoot apex on the release of dormant axillary buds located below. In the classical Thimann and Skoog (1933) experiment it was demonstrated that auxin applied on the decapitated stump of *Vicia faba* was able to inhibit this outgrowth and auxin was the first hormone suggested to play a key role in the apical dominance process. Auxin mainly originates from the shoot apex and does not enter buds to inhibit their outgrowth, so it was hypothesized to inhibit bud outgrowth indirectly (Snow, 1937; Morris, 1977). The precise auxin mode of action in this particular process is still under debate (see below) but it appeared that other signals were likely acting downstream of auxin.

Later, genetic approach was used to identify genes controlling axillary bud outgrowth and to understand the mechanisms involved in plants kept intact with their shoot apex. Specific screenings for high shoot branching without major defects in plant growth have been performed in mutant lines of pea [*ramosus* (*rms*)], *Arabidopsis* [*more axillary growth* (*max*)], rice [*dwarf* (*d*)], and petunia [*decreased of apical dominance* (*dad*)]. They have led to the isolation of the strigolactone (SL)-deficient and SL-response mutants. (Beveridge et al., 1996, 1997, 2009; Napoli, 1996; Stirnberg et al., 2002; Sorefan et al., 2003; Booker et al., 2004, 2005; Snowden et al., 2005; Zou et al., 2005, 2006; Arite et al., 2007, 2009). These mutants were first characterized by a bushy phenotype and reduced stature indicating that SLs are one of the key signals controlling shoot branching. Since the discovery of this novel class of plant hormone in 2008 (Gomez-Roldan et al., 2008; Umehara et al., 2008), progress in deciphering its signaling pathway has been very fast. The SL-receptor, an αβ-hydrolase, corresponding to the petunia *DAD2* and the rice *D14* genes, has been identified. It interacts with the F-box protein MAX2/D3/RMS4 (Arite et al., 2009; Hamiaux et al., 2012; Kagiyama et al., 2013) to mediate SL responses presumably targeting proteins to the proteasome for degradation. Several candidate target proteins of the SL-signaling pathway have been identified. In particular, the rice D53 protein activates shoot branching; it belongs to the small family of eight SMXL (SUPPRESSOR OF MAX2) proteins in *Arabidopsis* with weak homology to HEAT SHOCK PROTEIN 101 in the class–I Clp ATPase family (Jiang et al., 2013; Stanga et al., 2013; Zhou et al., 2013). These breakthroughs on SL biosynthesis and signaling pathways are described and discussed in several recent reviews (de Saint Germain et al., 2013a; Bennett and Leyser, 2014; Seto and Yamaguchi, 2014; Smith and Li, 2014; Waldie et al., 2014).

Nevertheless, SL mutants keep responding to environmental signals such as the photoperiod or planting density, or to removal of the shoot apex. Therefore other pathways than SLs’ influence shoot branching (Beveridge et al., 2003; Ferguson and Beveridge, 2009). Similarly, many other branching mutants have been identified, often because they were strongly affected in another trait (Murfet and Reid, 1993). Flowering genes, in particular those controlling the photoperiod response, also have a strong influence on basal shoot branching (Lejeune-Henaut et al., 2008). For instance, pea genotypes highly responsive to the photoperiod [*Hr* corresponding to *EARLY FLOWERING 3* (*EFL3*; Weller et al., 2012)] have typical morphological characteristics of winter-adapted plants with profuse branching and a rosette-type growth during the winter period (Lejeune-Henaut et al., 2008; Klein et al., 2014). Dwarfism is also often associated with increased shoot branching, hence genes regulating internode elongation, in particular those related to gibberellin (GA), affect shoot branching (Murfet and Reid, 1993; Silverstone et al., 1997; Lo et al., 2008). In most cases, it is not clear whether the effect on shoot branching is SL-dependent, but genetic analyses sometimes indicate independent pathways. For example, the pea *LE* gene controls GA biosynthesis and double mutants, SL (*rms1*) and GA (*le*) deficient, are more branched than single mutants (de Saint Germain et al., 2013b). Similarly, genetic analyses suggest SL-independent pathways for the *RAMOSUS6* (*RMS6*) and *RMS7* genes. The *rms6* and *rms7* mutants only display increased basal branching, including at cotyledon nodes for *rms6*. The additive branching phenotype of the double mutants (*rms6 rms7*, *rms6* or *rms7* associated with SL-related mutations) suggests that they may play a part in different pathways and are SL-independent (Rameau et al., 2002; Morris et al., 2003; Murfet, 2003; Ferguson and Beveridge, 2009). Interestingly branching pattern (basal, aerial, rosette type) and morphology (branch angle, width, number of branches *per* node) differ according to the gene involved. This could reflect the presence of independent regulatory networks.

In this review, recent advances in our understanding of how endogenous, environmental and developmental pathways control axillary bud outgrowth will be presented. Among the numerous environmental factors that can influence shoot branching, we focused on shade, an interesting and well-studied example of how light regulates the fate of axillary buds. These pathways may converge to common targets, so we propose key integrators of branching pathways. Last of all, we present how modeling/system biology can help to better understand and integrate these pathways.

## DIVERSITY OF THE ENDOGENOUS FACTORS AND MOLECULAR ACTORS THAT CONTROL SHOOT BRANCHING

Two models, involving either signals acting downstream of auxin or the “auxin canalization” process, may explain the indirect role of auxin in the control of apical dominance (Sachs and Thimann, 1967; Morris, 1977; Bangerth, 1994; Li et al., 1995; Li and Bangerth, 1999). These models were revisited and discussed with the discovery of (i) SLs as a novel class of plant hormones, and (ii) the molecular mechanisms involved in the regulation of polar auxin transport (PAT; Bennett et al., 2006; Dun et al., 2006; Brewer et al., 2009; Crawford et al., 2010). Below are discussed the different hypotheses on the indirect role of auxin in the control of bud outgrowth in interaction with SLs and cytokinins (CKs) together with recently published works indicating possible other pathways and mechanisms of control of bud outgrowth. In particular, we introduce sugars as the first signal triggering bud outgrowth in the process of apical dominance in pea.

### AUXIN ACTS UPSTREAM OF STRIGOLACTONES (SLs) AND CYTOKININS (CKs)

In the classical model, auxin controls the level of a root-to-shoot moving signal that enters axillary buds and regulates their outgrowth (Sachs and Thimann, 1967). The auxin signal is relayed by several downstream messengers such as CKs, ABA (Tucker and Mansfield, 1971; Cline, 1991), and SLs (Brewer et al., 2009).

A role for CKs in bud outgrowth emerged decades ago when direct CK applications onto dormant buds promoted bud outgrowth (Wickson and Thimann, 1958; Sachs and Thimann, 1967). ISOPENTENYL TRANSFERASE (IPT) enzymes control a rate-limiting step in CK biosynthesis, and transcript levels of *IPT* genes are modified in response to auxin levels. Repression of CK biosynthesis genes by auxin is commonly known (Miyawaki et al., 2004; Nordstrom et al., 2004; Tanaka et al., 2006). In the apical dominance context, the two pea *PsIPT1* and *PsIPT2* genes are rapidly up-regulated in the nodal stem after decapitation. CK quantifications in nodal stems and axillary buds 3 and 6 h after decapitation of pea plants suggest that CK biosynthesis first increases in nodal stem tissues, not in axillary buds. Then CKs are supposed to be transported into dormant buds to stimulate their outgrowth (Tanaka et al., 2006). This increase is not observed when auxin is applied to the cut surface of the decapitated plants. Moreover, in pea, a strong correlation between transcript levels of *IPT* genes at a given node and bud outgrowth at the same node was observed across a range of experiments and techniques used to decrease nutrient supply and auxin levels at the node level [decapitation, auxin transport inhibitor naphthylphthalamic acid (NPA) application, girdling, defoliation…; Ferguson and Beveridge, 2009]. By contrast, expression levels of SL-biosynthesis and auxin-responsive genes are not always well correlated with bud outgrowth phenotype. CK applications indicate that even when auxin and SL levels are very low, buds may not be able to grow because CK biosynthesis is limiting (Ferguson and Beveridge, 2009). Although the role of CKs in promoting bud outgrowth has been known for decades, their precise mode of action still remains unclear. In rice and pea, CKs down-regulate the *FINE CULM1/PsBRANCHED1* (*FC1/PsBRC1*) gene specifically expressed in axillary buds (Minakuchi et al., 2010; Braun et al., 2012). The TEOSINTE BRANCHED1, CYCLOIDEA, PCF (TCP) transcription factor FC1/PsBRC1 acts as a negative regulator of shoot branching and as an integrator of multiple pathways (Aguilar-Martinez et al., 2007; see below). In pea, CKs also appear to act independently of PsBRC1 because the *Psbrc1* mutant responds to CK application (Braun et al., 2012).

In several species, auxin up-regulates genes encoding two carotenoid cleavage dioxygenases (CCD7 and CCD8; Foo et al., 2005; Johnson et al., 2006; Zou et al., 2006; Arite et al., 2007; Hayward et al., 2009). CCD7 and CCD8 convert together with the β-carotene isomerase D27, all-*trans*-*β*-carotene into carlactone, a key intermediate in the SL biosynthesis pathway (Lin et al., 2009; Alder et al., 2012). Direct quantifications of SL levels are still needed to confirm this regulation by auxin. In pea, but not in rice, SLs up-regulate *PsBRC1* without any *de novo* protein synthesis, suggesting that *PsBRC1* is an SL-primary response gene (Dun et al., 2012). The way SLs regulate the transcription of *PsBRC1* still remain unknown, in particular the role in the control of shoot branching of the D53/SMXL proteins which are targeted for degradation in the SL pathway.

In this model, auxin controls CK and SL biosynthesis (Foo et al., 2005; Shimizu-Sato et al., 2009). Both hormones act downstream of auxin and converge to BRC1 to control bud outgrowth. CKs promote the process and SLs repress it. In *Arabidopsis* ABA signaling was recently shown to be stimulated in the axillary bud by BRC1 in shade conditions (see below; Gonzalez-Grandio et al., 2013). Therefore the different signals proposed to act downstream of auxin have a role in regulating bud outgrowth but the precise cascade of molecular events has still to be discovered.

### AUXIN ACTS DOWNSTREAM OF SLs: THE AUXIN CANALIZATION MODEL

The second hypothesis to explain the indirect action of auxin in bud outgrowth inhibition without entering the bud is based on the assumption that auxin export from a bud to the stem is necessary for bud outgrowth (Li and Bangerth, 1999). In the 1960s, by studying the formation of vascular strand networks, Sachs (1968) made the observation that exogenous application of auxin induces formation of new vascular strands oriented away from the applied auxin and toward already differentiated vascular tissue. He also observed that differentiated vascular tissue which is well supplied with auxin inhibits rather than attracts the formation of new vascular strands in its vicinity. Sachs (1975) proposed the auxin canalization model where auxin flow, starting by cell to cell diffusion, created a PAT system whereby the auxin flux was canalized in narrow files of cells from the leaves to the roots. Auxin itself polarizes and reinforces its own polar transport in these cells by some kind of positive feedback loop to form mature vasculature (Sachs, 1975). This hypothesis was later confirmed at the molecular level (Paciorek et al., 2005). Pre-existing strands of cells will behave as auxin sinks for new streams of auxin produced by young leaves but can also inhibit the connection of new vascular files when auxin levels and/or drainage are not sufficient in the pre-existing vascular strands. PIN (PIN-FORMED) proteins are essential components of cellular auxin efflux and polarity of their subcellular localization controls the direction of the auxin flow between cells (Petrasek et al., 2006). These proteins cycle rapidly between the plasma membrane (PM) and endosomes and their movement is highly regulated (Grunewald and Friml, 2010; Habets and Offringa, 2014). Sauer et al. (2006) suggested that the feedback regulation between auxin and PIN polarization is a mechanism responsible for auxin canalization.

To apply the canalization model to the control of shoot branching, competition between the main shoot apex and axillary buds for access to a common PAT stream (PATS) was suggested (Bennett et al., 2006; Crawford et al., 2010). Once exported from the main shoot apex (and from other growing shoots) and transported basipetally through the main PATS, auxin and/or its flux could regulate the establishment of canalized auxin transport from the axillary bud, and allow for its outgrowth. This model is based on the analysis of auxin transport in the SL-deficient high branching *Arabidopsis max* mutants that display high accumulation of PIN proteins at the PM and high auxin transport with high auxin levels moving through the PATS. In this model, SLs are thought to act upstream of auxin by modulating PAT in the main shoot (Bennett et al., 2006; Prusinkiewicz et al., 2009; Crawford et al., 2010; Shinohara et al., 2013). At the cellular level, SLs promote the removal of the auxin export protein PIN1 from the PM, therefore SLs diminish PATS in the main shoot and possibly also in the axillary bud, reducing its chances to outgrow (Shinohara et al., 2013).

At the plant phenotype level, this effect of SLs on PAT is characterized by increased competition among branches. This competition between shoot apices to export their auxin into the main PATS may also explain the process of correlative inhibition which has been largely studied using two-shoot plants: when a dominating, actively growing shoot reduces or inhibits the growth of a dominated shoot, the dominant branch has a higher capacity to transport labeled auxin and exports more endogenous IAA out of its apex than the dominated shoot. If the dominant branch is decapitated, growth rapidly resumes in the dominated branch, together with a strong increase in its capacity to transport IAA (Li and Bangerth, 1999). It appears that a dominating shoot displaying high auxin export has a strong inhibiting effect on the growth of other shoots. This is in apparent contradiction with what happened in an SL-deficient background where high PAT also occurred in the main stem but with a weak inhibiting effect on other shoots. Several questions need to be further studied to disentangle the web of these apparent contradictions. For example, we can still wonder (i) if correlative inhibition in two-shoot plants involves the same mechanism as described for the inhibition of PAT and axillary bud outgrowth by SLs, or (ii) if SLs act at an early stage of bud outgrowth or just after outgrowth has been triggered. In the same vein, we can wonder whether SLs also act on PIN proteins in the axillary bud/branch to inhibit auxin canalization and export from the bud, or how the PATS of the different branches interact, and how their mutual attraction and inhibition are regulated to form the global shoot branching pattern of a plant. These processes of auxin canalization and auxin transport via PIN proteins are intensively studied and tested by modeling approaches (Prusinkiewicz et al., 2009; Renton et al., 2012; Bennett et al., 2014, and see below) to understand these apparent contradictions. But despite major progress, the molecular processes involved are still not well understood, in particular how auxin in the PATS is able to attract or to inhibit auxin export from axillary bud is still not known (Bennett et al., 2014).

The two processes that may explain the indirect role of auxin in shoot branching (the downstream messengers and the auxin canalization model) have been discussed and debated in several reviews (Dun et al., 2006; Domagalska and Leyser, 2011; Waldie et al., 2014). They are not mutually exclusive and they are most probably both involved in the regulation of shoot branching (Waldie et al., 2014), perhaps at different stages of bud outgrowth. In pea, the *Psbrc1* mutant only has one long basal branch and very short aerial branches at the upper node level whereas *rms* mutants have one or two branches at most nodes (Braun et al., 2012). This higher shoot branching pattern of SL-deficient mutants in comparison to *Psbrc1* could be explained by the combination of both SL effects in the SL-deficient mutant *rms1* (low levels of *PsBRC1* transcripts in axillary buds and PAT affected). It would be of great interest to confirm whether the *Psbrc1* mutant has a normal PAT or not.

### GIBBERELLINS REPRESS SHOOT BRANCHING AND BRASSINOSTEROIDS STIMULATE IT

Gibberellins are well known for their role in internode elongation, transition from the vegetative phase to the floral phase and seed germination (Davies, 2007), but their role in shoot branching has barely been characterized. In *Arabidopsis* (Silverstone et al., 1997), rice (Lo et al., 2008), and pea (Murfet and Reid, 1993), GA-deficient mutants displayed higher shoot branching than the wild types. Conversely, recessive DELLA protein mutants such as the tomato *procera* mutant (Bassel et al., 2008) – DELLA proteins are main repressors of GA signaling – exhibited reduced shoot branching and/or altered branching patterns. Overexpressing GA catabolism genes to reduce GA levels produced increased branching phenotypes (Agharkar et al., 2007; Lo et al., 2008). In pea, GA- and SL-deficient double mutants displayed stronger branching than single mutants, suggesting that GAs act independently of SLs to repress branching (de Saint Germain et al., 2013b). But the rice DELLA SLR1 protein was recently proved able to interact with the D14 SL-receptor in an SL-dependent way. DELLA proteins lack typical DNA-binding domains and can bind to different classes of proteins, especially transcription factors involved in other pathways, and thereby inhibit their function (Daviere and Achard, 2013). *Arabidopsis* DELLA proteins were recently found able to bind to several class I TCP proteins at the shoot apex to regulate plant height (Daviere et al., 2014). By binding to the DNA-recognition domain of TCP transcription factors, DELLA proteins prevent them from activating cell cycle genes. Further studies should investigate a possible binding between BRC1 and DELLA proteins to identify a novel, GA-dependent and SL-independent mechanism that could explain the higher branching of GA-deficient mutants.

Dwarfism is not always correlated with increased branching. BR-deficient pea and rice mutants, unlike GA-deficient mutants, exhibit reduced branching (Murfet and Reid, 1993; Tong et al., 2009). In *Arabidopsis*, *bes1*-D, a gain-of-function mutant in bri1-EMS-suppressor 1 (BES1), a positive regulator of the brassinosteroid (BR) signaling pathway (Yin et al., 2002), displayed higher branching from the rosette whereas BES1-RNAi lines were less branched than the WT. Moreover the *bes1*-D mutant did not respond to GR24 treatment. BES1 and other homologs can interact with MAX2. This interaction promotes BES1 ubiquitination and degradation by the 26S proteasome and this degradation is regulated by SLs. Genetic analysis strongly suggests that BES1 functions downstream of MAX2 to inhibit SL signaling and promote shoot branching (Wang et al., 2013).

### SUGARS, NEW PLAYERS IN THE CONTROL OF SHOOT BRANCHING?

Sugars are a major source of carbon and energy and this aspect of their impact on branching is described below as “the trophic hypothesis.” Small sugars have also a signaling role in many physiological processes (Smeekens et al., 2010; Granot et al., 2013).

From a trophic point of view, axillary buds are regarded as sink organs that need to import sugars to meet its metabolic demand and support its growth. The bud capacity to grow can be reflected in their sink strength which represents its ability to acquire and use sugars. Therefore, in order to sustain its outgrowth, bud has to compete for sugars which constitute its main source of carbon and energy. In accordance with the trophic hypothesis, bud outgrowth is concomitant with (i) starch reserve mobilization in stem tissues, mostly in perennial plants (Alaoui-Sossé et al., 1994; Decourteix et al., 2008), (ii) high activity of sugar-metabolizing enzymes (Maurel et al., 2004; Girault et al., 2010; Rabot et al., 2012), and (iii) increased sugar absorption in bud (Marquat et al., 1999; Maurel et al., 2004; Decourteix et al., 2008), and PM H^+^-ATPase activity (Gevaudant et al., 2001; Alves et al., 2007) that creates an electrochemical gradient required for H^+^/nutrient co-transport (Pedersen et al., 2012). Parallel to this, soluble sugar content in buds (Marquat et al., 1999; Girault et al., 2010) and in xylem sap (Maurel et al., 2004; Decourteix et al., 2008) is increased. Moreover, this need for sugar is in line with the inhibition of bud outgrowth upon defoliation in sorghum (Kebrom et al., 2010) or sucrose diversion at the expense of buds in wheat *tin* mutants (tillering inhibition; Kebrom et al., 2012). It suggests that trophic competition for sugars among buds may be the possible cause of the precedence of certain buds over others along the same axis in walnut tree (Bonhomme et al., 2010). Auxin from the growing shoot apex might direct nutrient transport to the apex at the expense of the inactive lateral buds (Cline, 1991). In line with this, exogenous application of auxin to isolated nodes of *Rosa* sp. down-regulated the transcript levels of *RhSUC2*, a gene encoding a sucrose transporter highly expressed in outgrowing buds. Therefore auxin may deprive buds of their sucrose supply (Henry et al., 2011). Still, all these findings still need to be confirmed in other plant taxa, including herbaceous and perennial ones.

**FIGURE 1:**
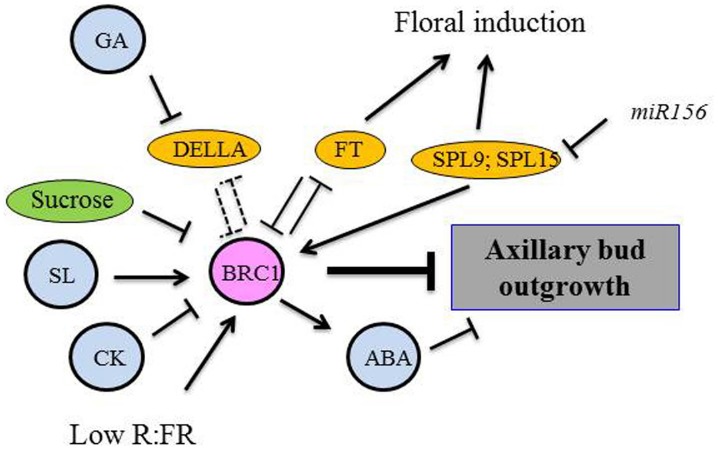
**Proposed model for BRC1 as an integrator of different pathways controlling axillary bud outgrowth.** Dashed lines, hypothetical protein–protein interactions.

The role of sugar as an early signal triggering bud activity has been suggested recently. Mason et al. (2014) demonstrated that sugars, unlike auxin, initiated the outgrowth of the basal bud in pea after shoot decapitation. Morris et al. (2005) proposed, for this system, the existence of an auxin-independent “fast-decapitation signal,” thought to trigger bud outgrowth after decapitation because bud release was observed before auxin depletion in the adjacent node and also in decapitated plants supplied with auxin on the decapitated stump. Thanks to time-lapse video, significant bud growth (nearly 0.1 mm) 2.5 h after decapitation even in buds located more than 40 cm from the decapitation site was evidenced (Morris et al., 2005; Mason et al., 2014), while a measurable growth was detected only 8 h after decapitation in former studies (Stafstrom and Sussex, 1992). Mason et al. (2014) demonstrated that the fast-decapitation signal was very likely sucrose that can move very fast in the plant (150 cm h^–1^) compared to auxin (1 cm h^–1^). The timing of axillary bud outgrowth matched well with their supply with phloem-transported photoassimilates; artificially increased sucrose levels promoted bud outgrowth in non-decapitated plants. Artificially applied sucrose down-regulated *BRC1* expression within the first 2 h of incubation in pea (Mason et al., 2014). This finding supports that sugar availability may play a significant part in the network mechanism related to shoot branching (Figure 1). The effect of auxin application to the decapitated stump to inhibit subsequent bud growth was observed only 20 h after decapitation suggesting that auxin, together with SLs and CKs, acted at a later stage.

All these data bring about new questions, i.e., whether sugar acts as trophic entity or as both a trophic and a signaling entity, and how buds perceive sugar (sucrose or hexose) availability and transduce the sugar signal. Preliminary data in isolated nodes of *Rosa* sp. suggest a role of the disaccharide signaling pathway in bud outgrowth, as sucrose or palatinose (a non-metabolizable sucrose analog) promoted bud outgrowth and expression of *RhVI1*, *Rosa hybrida* vacuolar invertase (Rabot et al., 2012). We can therefore wonder whether the sugar signal is conveyed through a cross-talk with the main hormonal network (auxin, CKs, and SLs) of shoot branching. The identification of certain sugar sensing/signal transduction components in meristem tissues (Pien et al., 2001; Halford and Paul, 2003) cannot rule out the dual role of sugars in bud outgrowth.

## EXAMPLES OF ENVIRONMENTAL AND DEVELOPMENTAL PATHWAYS INVOLVED IN THE CONTROL OF SHOOT BRANCHING

### DIVERSITY OF THE LIGHT-RELATED PARAMETERS THAT REGULATE SHOOT BRANCHING

Light as an energy source and a signal is a major environmental factor controlling branching, as recently reviewed by (Leduc et al., 2014). Different light-related parameters are sensed by plants and modulate bud outgrowth and branch development. Increasing light intensity, for example often promotes bud outgrowth and shoot elongation in herbaceous and tree species (Bahmani et al., 2000; Kawamura and Takeda, 2002; Niinemets and Lukjanova, 2003; Evers et al., 2006; Girault et al., 2008), while perception of light quality by plants brings them important information on the presence of competitive plants in their vicinity or on time of the year/the day. This helps them avoid or adapt to shade, prepare for seasonal changes and adjust their circadian clock (Facella et al., 2008; Kidokoro et al., 2009; Nakamichi, 2011; Staiger and Green, 2011). Such light signals are perceived by several types of photoreceptors [phytochromes (PHY), cryptochromes (CRY), phototropins (PHOT), zeitlupe (ZTL), flavin-binding Kelch (FKF1), and LOV kelch (LKP2/FKL1) proteins in plants; Li et al., 2011; Liu et al., 2011]. The understanding of the light transduction pathways and of the light/endogenous factor interactions in the control of branching is currently poor. The most studied process is the shade-avoidance-syndrome (SAS) induced by low red/far-red ratio (R/FR). It is discussed below. The impacts of other light conditions on the molecular control of branching have been discussed recently (Leduc et al., 2014).

### THE SHADE AVOIDANCE SYNDROME

In a canopy, capture of part of the incident light spectrum by plant green tissues reduces light intensity, R/FRs and blue light (B) intensity in the light transmitted further down (Smith, 1982). According to their ecology, plants located lower in the canopy may either adapt to this shade condition or try to avoid it (Smith, 1982). Most shade-avoiding plants display reduced branching and enhanced apical growth that help them compete for incident light (Casal et al., 1986; Ballaré and Casal, 2000; Franklin, 2008). This growth response is called the SAS.

Phytochrome B (PHYB) plays a major role in the sensing of R/FR in plants. Inactivation of the active Pfr form of PHYB by high FR, as in shade conditions, triggers SAS, and branching inhibition ensues (Finlayson et al., 2010; Kebrom et al., 2010; Gonzalez-Grandio et al., 2013). The contribution of other plant photoreceptors to SAS has been little investigated, even less the blue light photoreceptor CRY. Blue light alone can indeed trigger bud outgrowth as efficiently as white light (Abidi et al., 2013), suggesting that low blue light intensity, as under shade, may also be a signal that contributes to SAS-reduced branching.

Little is presently known about the molecular actors of R/FR signaling down to branching regulation. PHYB signals are transduced by BRC1. PHYB activation under high R/FR light down-regulated the transcriptional activity of a *TB1* homolog in sorghum (Kebrom et al., 2006, 2010) and of *BRC1* and *BRC2* in *Arabidopsis* (Finlayson et al., 2010). Conversely, increasing FR light promoted *BRC1* expression and thus contributed to reduced branching under shade (Gonzalez-Grandio et al., 2013). Interestingly, *BRC2* transcript levels remained unchanged in this condition, suggesting a different role than *BRC1*. The study of *brc1* mutants’ transcriptional profiles under simulated shade showed that several photosynthesis, cell cycle, and protein synthesis genes were repressed by *BRC1* and ABA-related genes were up-regulated by *BRC1* (Gonzalez-Grandio et al., 2013). Therefore a multiple range of target mechanisms may be controlled by the shade signal downstream of BRC1. As these mechanisms are controlled by BRC1, they may not be specific to low R/FR. The presence of numerous TCP-binding sites in the promoters of the *BRC1*-down-regulated genes suggests direct transcriptional regulation of these genes by the TCP transcription factor. In the case of ABA, *BRC1* is thought to promote the transcription of ABA-responsive regulators such as *ABF3* and *ABI5*. The ABA biosynthesis mutants *nced3-2* and *aba2-1* exhibited enhanced branching capacity under low R:FR (Reddy et al., 2013), so a direct role of ABA in the repression of bud outgrowth under shade may also exist.

Light may also interact with auxin in the regulation of shoot branching under shade. Auxin-responsive genes were up-regulated in stem segments of *phyB Arabidopsis* mutants. These mutants display constitutive reduced branching as in SAS (Reddy and Finlayson, 2014), suggesting that PHYB promotes branching through the repression of auxin signaling. Still, further investigations need to be carried out to decipher the exact role of auxin in shoot branching under low R/FR. In sorghum, inhibition of outgrowth in a *phyB* mutant and FR treatment were also correlated with a sharp increase in the transcript levels of the SL-signaling gene *SbMAX2* in buds (Kebrom et al., 2010). Regulation of *SbMAX2* by shade may be SL-independent as in *Arabidopsis*, where *max2* mutation had pleiotropic effects compared to other *max* mutations. For example, the *max2* mutant was affected in seed germination and seedling de-etiolation whereas the SL-biosynthesis *max3* and *max4* mutants, were not, suggesting that MAX2 is involved in the regulation of seedling photomorphogenesis independently of SLs (Shen et al., 2012). Moreover, over-expression of *MAX2* in *Arabidopsis* SL-deficient mutants (*max1*, *max3*, *max4*) partially reduced their branching (Stirnberg et al., 2007) but the reason for that effect remains unclear (Waldie et al., 2014).

Shade is also characterized by decreased incident light intensity. Whether low light intensity contributes to SAS or whether SAS is primarily a response to the changes in light quality associated to shade has long been debated (Bartlett and Remphrey, 1988; Ballaré and Casal, 2000; Evers et al., 2006; Wubs et al., 2013, 2014). In fact, results in *Arabidopsis* suggest a fine tuning of shoot branching by these two parameters whereby reduced branching will only occur when R/FR and light intensity are both low (as under established shade), while branching will still be promoted when R/FR alone is low (as in the neighborhood of a not yet shading plant; Su et al., 2011). This interaction between responses to light intensity and R/FR is thought to allow plants to distinguish between the two environments (established shade vs. neighbor avoidance) and finely adjust their development. Important variations among species most probably exist. Interestingly, in *Arabidopsis*, light intensity is not believed to act on branching through the down-regulation of photosynthetic assimilation that indeed takes place in shade, but through an autonomous and different pathway from that triggered by low R/FR and PHYB-associated mechanisms. This pathway could involve interactions with growth regulators (Su et al., 2011).

### CROSS-TALK BETWEEN THE FLOWERING AND BRANCHING PATHWAYS

Shoot branching is strongly influenced by developmental processes such as flowering. This crosstalk between flowering and branching is complex as floral initiation and branching are both controlled by similar environmental (photoperiod) or endogenous (plant growth regulators) factors, suggesting common regulatory pathways between the two processes. Late-flowering mutants often exhibit modified branching patterns. In *Arabidopsis*, FLOWERING LOCUS C (FLC) and FRIGIDA (FRI), two floral repressors in the vernalization pathway (Andres and Coupland, 2012), also regulate stem branching (Huang et al., 2013). In forage pea lines, the dominant *HR/ELF3* allele is late flowering under short-day conditions. *HR* is associated with increased branching and winter frost tolerance (Lejeune-Henaut et al., 2008; Weller et al., 2012). Furthermore, floral initiation is marked by dramatic physiological modifications at the shoot apex. We hypothesize that these modifications modify hormone balance and transport leading to bud release. In several species, dormant axillary buds below the flowering node are frequently released from dormancy at floral transition. In *Arabidopsis* as in other species, the gradient of bud outgrowth reversed at floral transition with apical buds activated first after floral transition (McSteen and Leyser, 2005).

The *FT (FLOWERING LOCUS T)/TFL1 (TERMINAL FLOWER1)* gene family is involved in the control of floral induction, but also in plant architecture through the control of determinate and indeterminate growth (McGarry and Ayre, 2012; Pin and Nilsson, 2012). The floral activator FT and the floral repressor TFL1 are components of the florigen and anti-florigen pathways, respectively. FT is a mobile signal that promotes flowering in the shoot apical meristem by regulating the FD transcription factor (see Pin and Nilsson, 2012, for a review). The florigen pathway also stimulates shoot branching. Late-flowering *ft* mutants display delayed lateral shoot outgrowth and reduced lateral shoot growth rates (Hiraoka et al., 2012). The FT/TFL1 ratio might regulate the balance between different developmental processes in response to environmental cues, and the florigen pathway may fulfill the criteria for a plant growth regulator (Shalit et al., 2009). According to this hypothesis, the FT/TFL1 balance might be a regulator of branching: a high ratio leads to increased branching and a low ratio to decreased branching, as shown in rice (Tamaki et al., 2007), rose (Randoux et al., 2014), or tomato (Lifschitz, 2008) by using mutants or transgenic plants that over-expressed *FT/TFL1* genes.

A mode of action was recently proposed in *Arabidopsis*, where FT interacts with BRC1. In axillary buds, FT and TSF (TWIN SISTER OF FT, a paralog of FT) proteins interact with BRC1. The *brc1-2* mutant is highly branched, and its lateral branches flower earlier (Niwa et al., 2013). BRC1 might inhibit floral induction by interacting with FT/TSF. Conversely, we can hypothesize that FT stimulates bud outgrowth by interacting with BRC1 (Figure 1). Indeed, BRC1 and FT may neutralize each other by interacting together: in the case of the FT/BRC1 interaction, FD is not activated by FT (no flowering) and BRC1 is inactive (branching is possible). Further experiments are needed to test this hypothesis.

### THE SQUAMOSA PROMOTER BINDING PROTEIN-LIKE PATHWAY

Plant-specific SPL (SQUAMOSA BINDING PROTEIN LIKE) transcription factors control different aspects of plant development such as phase transitions (juvenile to adult and adult to floral), leaf development and plant maturation (Huijser and Schmid, 2011). Different members of the *SPL* gene family (10 out of 16 in *Arabidopsis*) are post-transcriptionally controlled by *miR156* (Rhoades et al., 2002). Overexpression of *miR156b* brought about a marked bushy phenotype with increased numbers of rosette leaves in *Arabidopsis* (Schwab et al., 2005; Wei et al., 2012). Double mutants obtained from the two *Arabidopsis SPL9* and *SPL15* paralogs displayed a less severe branching phenotype, suggesting that other *miR156*-targeted *SPL* genes also affect shoot branching (Schwarz et al., 2008). Interestingly, overexpression of *miR156b* additionally increased seed carotenoid content, so SL metabolism might also be affected (Wei et al., 2012). This hypothesis was demonstrated in potato where overexpression of *miR156* altered plant architecture and SL and CK contents (Bhogale et al., 2014).

In maize, the dominant natural mutation *Corngrass* is caused by the overexpression of two tandem *miR156* genes. Among others, the mutant displays a high tillering phenotype and a short stature. Seven of the 13 potential *SPL* targets of *miR156* were strongly down-regulated in the *Corngrass* mutant. In rice, accumulation of the SPL9/15 homolog, OsSPL14 or IDEAL PLANT ARCHITECTURE1 (IPA1) led to plants with fewer tillers, stronger productive stems, increased lodging resistance and increased yield. These results suggest that SPL proteins are branching inhibitors (Jiao et al., 2010). *IPA1/OsSPL14*, regulated by the microRNA *OsmiR156*, directly activates *OsTB1* (Jiao et al., 2010). Luo et al. (2012) think that SLs and OsSPL14 act in two independent pathways to control tiller growth because overexpression of *OsSPL14* results in reduced tillering in both the WT and the SL-deficient *d10* and SL-response *d3* mutants.

## KEY PLAYERS IN THE INTEGRATION OF BRANCHING PATHWAYS

### THE TCP TRANSCRIPTION FACTOR BRC1/TB1

The TCP transcription factor BRC1/TB1 is specifically expressed in axillary buds. It is considered as a common target for several endogenous and environmental signals at the transcriptional and post-transcriptional levels; therefore it could be a key integrator of different pathways involved in the control of bud outgrowth (Figure 1). In pea axillary buds, *PsBRC1* transcript levels are up-regulated by SLs and down-regulated by CKs and sucrose (Braun et al., 2012; Mason et al., 2014). FR treatment and a low R:FR ratio induced *BRC1* expression in *Arabidopsis* axillary buds and BRC1 was shown to be necessary for branching inhibition in response to shade. Multiple targets downstream of BRC1 have been identified in *Arabidopsis* under low R:FR ratio including promotion of ABA signaling, repression of cell proliferation and protein synthesis (Gonzalez-Grandio et al., 2013). DELLA proteins bind to several TCP proteins (Daviere et al., 2014), so the mechanism whereby GA represses shoot branching may be an interaction between DELLA proteins and BRC1 which is also a TCP protein. In the presence of GAs, DELLA proteins are degraded and BRC1 proteins are believed to be active (branching inhibition; Daviere and Achard, 2013; Daviere et al., 2014).

In grasses, a central role of TB1/FC1 has also been demonstrated, but with slight differences. In rice, while the expression of the *OsTB1/FC1* gene is down-regulated by CKs, it is not transcriptionally regulated by SLs. But SLs act at least partially *via* FC1, as the *fc1* mutant does not respond to SL application. *OsTB1/FC1* is also up-regulated by IPA1/OsSPL14 in an SL-independent manner (Luo et al., 2012). In maize, where domestication selected a gain-of-function allele of *TB1*, an SL-independent *TB1* sub-network has evolved to control branching (Guan et al., 2012).

### THE POLAR AUXIN TRANSPORT

**FIGURE 2:**
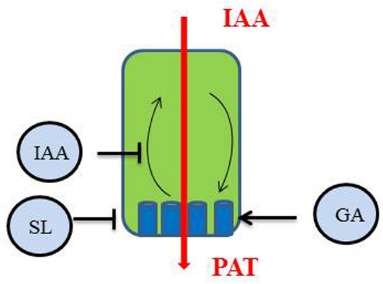
**Polar auxin transport (PAT) in the main shoot is regulated by different hormones through their action on the behavior of PIN1 proteins (blue) in the cell: IAA inhibits PIN endocytosis (Paciorek et al., 2005), SL trigger PIN1 depletion from the plasma membrane (PM; Shinohara et al., 2013), GA stabilize PIN proteins to the PM (Willige et al., 2011)**.

The directional transport of auxin is essential for most plant development processes and has been particularly investigated to explain the pattern of leaf initiation at the shoot apical meristem (phyllotaxy) or the pattern of leaf vascularization. PAT in the main stem is also an important component of the control of shoot branching possibly by preventing the establishment of PAT out of axillary buds. Research on PAT focuses on the behavior of PIN proteins in cells to understand how they can influence an overall process at the plant level (Grunewald and Friml, 2010). SLs, by rapidly stimulating PIN1 depletion from the PM (Shinohara et al., 2013), reduce auxin flux in the PATS (Crawford et al., 2010). PIN transcript levels, PIN protein levels at the PM and PIN localization within cells are tightly regulated by many environmental and endogenous factors (for an in-depth recent review, see Habets and Offringa, 2014). Consequently, SLs very likely are not the only regulators of PAT in the stem and PAT could be considered as another important integrator of endogenous signals in the control of shoot branching (Figure 2). Auxin itself stabilizes PIN proteins at the PM by inhibiting endocytosis of the constitutively cycling PIN proteins (Paciorek et al., 2005). As a result, auxin efflux is stimulated and its polar transport in the stem is enhanced. GAs also promote and/or stabilize the PM localization of PIN proteins (Willige et al., 2011). Interestingly, auxin transport in GA-deficient (*ga1*) and GA-response (*gid1a gid1c*, *gai*) mutants of *Arabidopsis* was reduced in inflorescence stems, with a sharp reduction in the abundance of PIN1 proteins compared to the wild type, but no change in PIN polarity (Willige et al., 2011).

## MODELING COULD BRING IN A BETTER UNDERSTANDING OF SHOOT BRANCHING REGULATION

As described above, the regulatory network of shoot branching involves multiple players of various types (plant development, genotype, hormones, nutrients) that interact with feedback loops at both bud and plant scale. This complexity is made even more intricate by the dynamics of the system related to plant development and the fluctuation of environmental variables. The functioning of complex and dynamic systems cannot be inferred by experiments alone, which provide only a picture of regulatory networks in particular situations and at particular times. One approach consists in combining experiments with modeling, which offers the possibility to position and link the multiple and heterogeneous players logically. In such an approach, knowledge and hypotheses about the branching regulatory network can be gathered into a model and the hypothetical part can be assessed by thorough comparisons between the behavior of the modeled system and actual plant behavior (Hofmann, 2009; Bongers et al., 2014; Chew et al., 2014).

Modeling can be combined to experiments to provide insights into branching regulation. By using modeling in parallel with stepwise laboratory research Dun et al. (2009) discovered new interactions in the pea *RMS* gene network. By modeling auxin transport canalization Prusinkiewicz et al. (2009) demonstrated that the control of bud outgrowth by an auxin transport switch was sufficient to qualitatively reproduce several branching behaviors in wild-type or mutant *Arabidopsis* plants. By calibrating auxin transport models on experimental data, Renton et al. (2012) demonstrated that the pattern of auxin transport observed in pea stems was not cogent with the assumptions that (i) auxin levels near the bud are the initial signal that triggers bud outgrowth, (ii) auxin flow is dependent on auxin concentration and limited by the amount of transporters. Finally, by modeling different hypothetical laws for bud growth response to R:FR, Evers et al. (2007) managed to analyze which model allowed to best reproduce tillering response to density.

**FIGURE 3:**
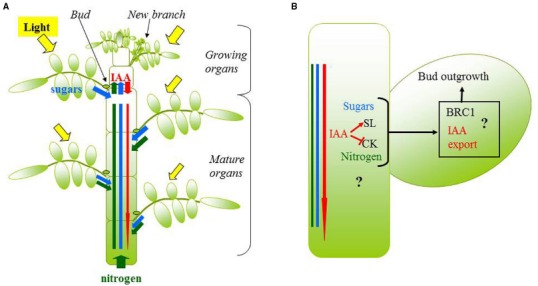
**Schematic representation of the main processes and players of bud outgrowth that should be represented in a model, consisted in a plant module (A) and a bud module (B).** In **(A)**, plant structure is explicitly represented and coupled with a light model to simulate the light perceived by each organ (in yellow). The level of sugar (blue rectangle), nitrogen (green rectangle) and auxin (IAA; red thin arrow) near each bud is the result of the production or assimilation by source organs (photosynthetic organs for sugars, roots or leaves for nitrogen, growing organs for auxin; large arrows), the utilization by sink organs (growing organs for sugars, nitrogen, and roots for auxin; large arrows), and possibly transport processes (red thin arrow). In **(B)**, the concentration of sugars, nitrogen, and auxin near a bud interact with cytokinins and strigolactones in the stem, to control bud outgrowth through key integrators in the bud such as BRC1 or auxin transport.

The above-mentioned modeling studies each focused on understanding the role of one player of bud outgrowth (auxin, SLs, or R:FR). Integrating the role of the various players into a single scheme remains a challenge. We propose the following representation of the branching regulatory system. Branching pattern is the result of the outgrowth dynamics of the different buds. Outgrowth timing of one specific bud depends on the dynamics of hormones, nutrients such as sugars, and possibly light signals locally perceived by the bud. Those dynamics are closely dependent on bud position in the plant (Chelle, 2005; Morris et al., 2005; Bonhomme et al., 2010), on the growth of other plant parts (e.g., newly formed leaves are a source of auxin and a sink for sugars), and environmental fluctuations. From this representation, research issues arise at two levels. At the bud scale, we need to unravel which are the players regulating bud outgrowth locally, what their relationships are, and how they control bud outgrowth together. At the plant scale, we do not know how plant growth characteristics (which may be proper to one genotype) and environment affect dynamically the levels of the various players near each bud of the plant, and what the consequences for the final branching pattern are. One particular question is how the development of a branch changes the hormonal, nutrient, and light state for the other buds of the plant. Some information is available in the literature to answer these questions and modeling can help, as described below (Figure 3).

Physiological studies have identified several hormonal players (CK, SL, IAA, GA, ABA) in bud regulation and the role played by sugars has recently been evidenced. All these players interact with one another (e.g., effect of auxin on CKs and SLs, effect of SLs on auxin transport; see above). However, much work is still needed to understand how the different players interact and control bud outgrowth. Interesting questions will be to distinguish between the trophic and signaling roles of sugar in bud outgrowth control, as well as distinguishing between bud outgrowth triggering and the subsequent bud elongation phase, which may involve different processes (Cline, 1997). To investigate bud outgrowth regulation and avoid the complexity of the whole plant system, it is possible to cultivate buds *in vitro*. This makes it possible to control bud local conditions and get rid of the dependence between buds and the rest of the plant (Chatfield et al., 2000; Henry et al., 2011; Rabot et al., 2012). In this system, associating measurements of bud growth and physiological state (e.g., auxin transport, gene expression) to modeling of the known and hypothetical physiological processes will be a promising step toward a better understanding of the regulatory network of bud outgrowth.

To assess how plant growth and environment affect the levels of the various players near each bud of a given plant, one approach can be to develop a functional–structural plant model. This modeling approach consists in representing plant physiological functioning in a realistic plant botanical structure (Prusinkiewicz and Lindenmayer, 1990; Evers et al., 2011; Evers and Vos, 2013). Each organ is individually represented and positioned in the plant, so that the hormonal, nutrient, or physical environments (e.g., light) can be estimated locally for each organ. Moreover, the different organs have topological connections between one another, so that the specific behavior of one distant organ (which may vary according to genotype or environment) can modulate nutrient and hormonal conditions near a bud. A model estimating the dynamics of local players at the level of each bud should simulate the temporal dynamics of: (i) nutrient uptake, consumption, and distribution within the plant, (ii) hormonal production, catabolism, and distribution, (iii) plant development and organ growth (which determines source and sink dynamics for sugar and auxin, for example), (iv) light capture by each organ (which determines its photosynthesis, nitrogen content, or possibly auxin production levels for example).

To estimate the amount of light intercepted by each organ, structural plant models are interfaced with light models. Different light models exist in the literature, with different accuracy levels (Chelle, 2005; Monsi and Saeki, 2005). The more detailed ones provide the amount of light intercepted at each point of a plant and a 3D representation of plant structure (Chelle and Andrieu, 1998). To estimate plant structure dynamics, a sufficient approach for our purpose would be to fit a model of plant development and organ growth on experimental data. Knowledge is currently not sufficient to model plant growth in a predictive way. Two models exist in the literature to estimate auxin distribution within a plant (Prusinkiewicz et al., 2009): simulated auxin production and auxin transport canalization to the roots, which accounts for the role of PIN polarization and its feedback regulation by the directional auxin flux. By contrast, Renton et al. (2012) demonstrated that auxin transport in pea was not limited by auxin transporters and could be simulated simply by assuming a constant propagation rate, indicating that auxin transport is independent of the auxin level. To understand bud outgrowth control, such auxin models have to be extended to account for the distribution of other hormones (e.g., CKs, SLs) within the plant, the possible impact of environment on hormone economy, as well as the effect of flowering.

Finally, several more or less detailed models are available in the literature to model the temporal dynamics of nitrogen and carbon compounds (Tabourel-Tayot and Gastal, 1998; Allen et al., 2005; Luquet et al., 2006; Kang et al., 2008; Fanwoua et al., 2014). In all these models, nitrogen and carbon dynamics are modeled from the difference between assimilation (photosynthetic organs for C, roots for N) and consumption by growing organs. However, they differ by their complexity levels. In the most simple approaches, a global nutrient status of the plant is calculated through indices such as the nitrogen nutrition index (NNI; Justes et al., 1994) or the ratio between the amount of reduced carbon compounds produced by source organs and the amount used by sink organs (e.g., Luquet et al., 2006). In more complex approaches, the conversion between different metabolic forms of nitrogen and carbon compounds is modeled (e.g., Bancal and Soltani, 2002; Minchin and Lacointe, 2005; Bertheloot et al., 2011). Thanks to this second approach, amino acid, nitrate or sucrose concentrations can be assessed. We believe that this approach is essential to understand bud outgrowth regulation because unlike indices, concentrations have a physical meaning. For this approach, one should decide what is the appropriate description degree for the processes. One recurring issue is the need to model transport processes. The answer probably highly depends on plant size. A detailed model could also help to decide what simplifications can be made a posteriori.

## CONCLUSION

In this paper, we highlighted the complex control of axillary bud outgrowth and of shoot branching and the interplay of several pathways in its control. Parts of the regulatory pathways have been identified, however, current knowledge does not provide a unified view of how environmental and developmental signals, as well as hormones, and nutrients controls are integrated to determine the branching pattern of a plant. Mason et al. (2014) suggested that bud outgrowth induced by decapitation in pea may be controlled at different stages by auxin and sugar: an increase in sugar availability would be the initial trigger by which the bud would move from a dormancy state to a release state, while auxin would act later in the bud outgrowth process by conditioning the transition to sustained growth. However, the role of sugar as the first signal triggering bud release remain to be demonstrated for contexts other than apical dominance. A key question is at which stage(s) of bud development the different pathways influence shoot branching. Several authors have suggested the idea of different stages in axillary bud development to emphasize a possible cycling between a dormant state (no visible sign of growth) and sustained growth (Stafstrom and Sussex, 1988; Shimizu-Sato and Mori, 2001; Beveridge, 2006). The sequential molecular events and the role of other signals than auxin and sugar also need to be deciphered. Modeling is an appropriate way to organize data and link the different players, and can help to formalize and assess assumptions about missing links. We propose a modeling approach, which considers the plant system at two levels: the level of the plant, which implies feedbacks from the rest of the plant (e.g., flowering, internode elongation,…) on the conditions perceived by the bud; and the level of the bud itself, which implies several players closely interlinked, to represent how bud responds to its environment. This approach aims to disentangle which behavior is specific to the bud outgrowth process from behavior being an indirect result of the impact of other plant parts. Obviously the full potential of the modeling approach will only be reached if modeling is conducted in parallel with experiments specifically designed to refine hypotheses and assess the proposed regulatory networks.

### Conflict of Interest Statement

The Guest Associate Editor Alexandra Jullien declares that, despite being affiliated at the same institution as the authors Catherine Rameau and Bruno Andrieu, the review process was handled objectively and no conflict of interest exists. The authors declare that the research was conducted in the absence of any commercial or financial relationships that could be construed as a potential conflict of interest.
